# Black/White Differences in Perceived Weight and Attractiveness among Overweight Women

**DOI:** 10.1155/2013/320326

**Published:** 2013-02-26

**Authors:** Taona P. Chithambo, Stanley J. Huey

**Affiliations:** Deptartment of Psychology, University of Southern California, 3620 S. McClintock Avenue, SGM 501, Los Angeles, CA 90089, USA

## Abstract

Numerous studies have reported that Black women are more satisfied with their bodies than White women. The buffering hypothesis suggests that aspects of Black culture protect Black women against media ideals that promote a slender female body type; therefore, Black women are expected to exhibit higher body esteem than White women. To test this hypothesis, the current study aimed to assess the influence of race on weight perception, perceived attractiveness, and the interrelations between body mass index (BMI) and perceived attractiveness among overweight and obese women. Participants were 1,694 respondents of Wave IV of the National Longitudinal Study on Adolescent Health (*M* = 28.89 years). Black (*n* = 531) or White (*n* = 1163) obese or overweight women were included in the current study. As expected, Black women reported lower perceived weight and higher attractiveness than White women, despite higher body mass for Black women. Furthermore, race moderated the relationship between BMI and perceived attractiveness; for White women, a negative relationship existed between BMI and attractiveness, whereas for Black women, BMI and attractiveness were not related. The study findings provide further support for the buffering hypothesis, indicating that despite higher body mass, overweight Black women are less susceptible to thin body ideals than White women.

## 1. Introduction

Black women are at heightened risk for obesity when compared to White women and tend to weigh more than White women on average [1]. Yet, despite higher body mass, research suggests that Black women are more satisfied with their bodies than White women [2]. Furthermore, Black women tend to attribute fewer negative qualities to overweight people than White women [3] and are less likely to engage in disordered eating behaviors to lose weight [4]. Powell and Kahn [5] suggested that Blacks are less susceptible to body dissatisfaction than Whites due to their adherence to cultural ideals that promote a heavier body type and discourage stringent body weight goals, a perspective labeled the buffering hypothesis [6, 7]. Empirical research provides some support for this assertion; on average, Black men prefer a heavier female body weight than White men, and the ideal body weight reported by Black women is higher on average than the ideal reported by White women [2]. Nonetheless, some scholars argue that the discrepancy between Black and White body preference is overstated; for example, a meta-analysis by Grabe and Hyde [6] concluded that though White women were more dissatisfied with their bodies than Blacks, the magnitude of the difference was small. Also, Cachelin and colleagues found that when age, body weight, and education are controlled for, ethnicity does not influence body shape preference or tolerance for obesity [8].

Though past research has identified racial differences in body image and weight perception [6], replication studies are needed to provide empirical support for the tenets of the buffering hypothesis. In addition, as health researchers are concerned that tolerance for heavier body types may contribute to high obesity prevalence in the Black community [2], it is important to elucidate the potential effect of race on the relationship between body weight and perceived attractiveness among overweight individuals. The current study examined this issue using survey data from 1,694 overweight or obese women. Based on past research, it was hypothesized that overweight Black women would exhibit higher body mass and report higher self-rated attractiveness than White women. Because Black women have been found to underestimate their body mass when compared to White women [9], we predicted that overweight Black women would report lower perceived weight than White women. Also, in accordance with the buffering hypothesis, we predicted that the relationship between body mass and self-rated attractiveness would be moderated by ethnicity; specifically, White women were expected to report lower attractiveness as their body mass index (BMI) increased, whereas BMI and attractiveness were expected to be unrelated among Black women. 

## 2. Methods

### 2.1. Participants

Survey respondents were participants in Wave IV of the National Longitudinal Study of Adolescent Health (Add Health), a publicly available database. Data collection for the study began in 1994-1995, when participants were in grades 7–12, with the aim of examining the influence of individual and environmental factors on health and health behavior. Participants were students of 80 randomly selected high schools in the United States. Assignment was stratified by region, school size, school type (public, private, and parochial), White/Black ethnicity, grade span, and curriculum. Feeder schools of students in 7th grade or higher were also selected for inclusion. Participants of Wave IV were aged 24–32. Ninety-minute interviews were conducted in the participants' homes, with respondents completing sensitive materials using a computer-assisted self-administration procedure. Research personnel administered less sensitive items with computer assistance, conducted physical measurements, and collected biospecimens. The current study included Black and White women who were overweight or obese (BMI > 25; [10]). Detailed information regarding data collection procedures is available elsewhere [11]. 

### 2.2. Measures

#### 2.2.1. BMI

Study staff obtained measurements of participants' height and weight using standardized procedures, and BMI was calculated on the basis of these measurements following Centers for Disease Control and Prevention guidelines [10]. 

#### 2.2.2. Race

Because self-report race data were not available, race was derived from assessors' report of the respondents' ethnicity; participants in the current study were identified as either “White” or “Black or African American.” 

#### 2.2.3. Weight Perception

Using a 5-point scale, participants were asked to respond to the survey question “How do you think about yourself in terms of weight?”, with 1 indicating “very underweight” and 5 indicating “very overweight.”

#### 2.2.4. Self-Rated Attractiveness

Participants responded to the survey question “how attractive are you?” on a 1 to 4 scale, with 1 indicating “very attractive” and 4 indicating “not at all attractive.” Responses were reverse-coded so that a higher score would correspond with increased attractiveness.

#### 2.2.5. Education Level

Because past research has implicated socioeconomic factors in explaining race differences in body dissatisfaction [8], education level was included in regression analyses as a covariate. Education was assessed on a 1 to 12 scale, with 1 denoting “8th grade or less” and 12 indicating “completed postbaccalaureate education.” 

### 2.3. Analyses

Independent samples *t*-tests were run to evaluate mean group differences in BMI, weight perception, and perceived attractiveness. Hierarchical regression analyses were used to evaluate the moderation hypothesis, with perceived attractiveness as the dependent variable. Age and education level were included as covariates, as past research has identified both variables as predictors of body dissatisfaction [12, 13]. Age and participant race were included in Step 1 of the regression analyses, with perceived attractiveness as the dependent variable. In Step 2, BMI was added to the model. In Step 3, a Race × BMI interaction term was included. Finally, to determine whether socioeconomic factors influenced these relationships, education level was included in the final step of the model.

## 3. Results

The sample was 28.89 years old on average (SD = 1.74). Sixty-nine percent of respondents were White (*n* = 1163) and 31% were Black (*n* = 531). The mean BMI for the sample was 32.92 (SD = 6.59), well above the cutoff for overweight. 

Results for mean group differences are displayed in Table 1. On average, Black women were heavier than White women. Yet, Black women reported lower perceived weight than White women. Also, as we predicted, Black women perceived themselves to be more attractive when compared to White women. Thus, although Black women were significantly heavier than their White counterparts, they actually perceived themselves as being less overweight and more attractive.

**Figure 1:**
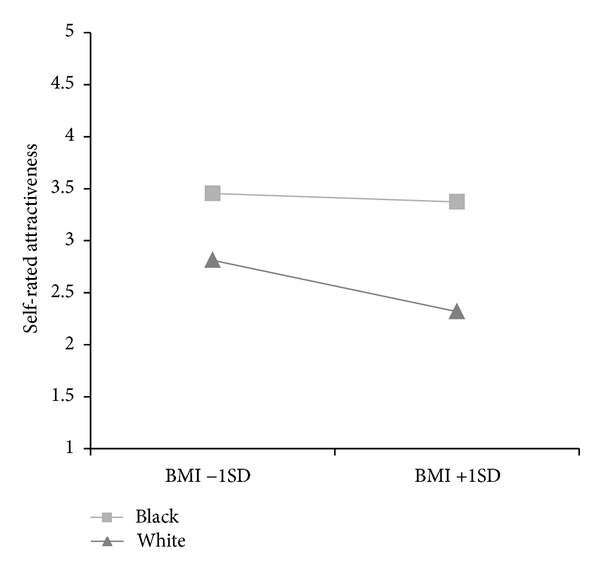
Attractiveness means by race and BMI.

Hierarchical regression analyses are summarized in Table 2. Steps 1 and 2 revealed that BMI was a significant predictor of self-rated attractiveness, with higher BMI associated with lower perceived attractiveness; also, Black women perceived themselves to be more attractive than White women. Moreover, a significant interaction was found between BMI and race; for White women, a negative relationship was detected between BMI and self-rated attractiveness (*β* = − .15, *t* = − 7.52, *p* < .001) while no relationship was found for Black women (*β* = − .01, *t* = − .23, *p* = .82). Both the Step 2 BMI term and the Step 3 BMI × Race interaction term contributed significantly to explaining variance in the model, as indicated by the *R* ^2^ change values. Education level was not a significant predictor of perceived attractiveness, nor did inclusion of the variable significantly improve the model. Group means for attractiveness at a standard deviation above and below the mean are displayed in Figure 1 [14].

## 4. Discussion

These results support the buffering hypothesis and findings from past research [6, 7], as overweight Black women rated themselves more attractive and reported lower perceived weight than White women despite higher body mass. Furthermore, ethnicity moderated the relationship between BMI and perceived attractiveness. BMI and attractiveness were negatively associated for White women, while no association was found for Black women. Thus, it can be concluded that for Black women, appearance evaluation is independent of weight, while White women experience less satisfaction with their appearance as their weight increases.

 It is possible that cultural differences between Blacks and Whites contributed to the observed results, as postulated by the buffering hypothesis. It has been suggested that Black Americans associate thinness with poor health and economic instability, while more curvaceous body types are indicative of health and beauty [2]. Also, previous studies have found that Black women are less likely to idealize the thin body type commonly portrayed in mainstream American media [15]. Black women report that media representations of the thin ideal are more relevant to Whites [2], contributing to lower drive for thinness and body dissatisfaction among Black women [6, 15].Furthermore, African Americans who report high other-group orientation report higher body dissatisfaction than African Americans oriented to the values of their own cultural group, suggesting that African American values serve a protective role against body dissatisfaction [7].

Though past research holds that Black cultural ideals buffer them from body image disturbances, less is known about the specific cultural traits that foster positive body esteem. Past research posits that Black women assess their appearance in broader terms, such as carrying oneself with confidence and wearing fashionable hairstyles, makeup, and clothing [2, 16]. This may render Black women less susceptible to media influences that define female beauty primarily on the basis of thinness. More research is needed to elucidate precise cultural values that contribute to high body satisfaction among Black women.

As health researchers have expressed concern that positive body images among overweight Black women may contribute to elevated obesity in the Black community, these findings have implications for weight intervention with this population. Because Black women are less likely to associate overweight with reduced attractiveness, future interventions for obesity among Black populations may benefit from promoting health and wellness as primary advantages of weight loss, rather than focusing on enhanced physical appearance. As body dissatisfaction is a predictor of poor psychological outcomes [17–19], it is of primary importance that obesity interventions for Black individuals aim to reduce body weight without instilling cultural perspectives that idealize a slender body type. 

Though the current study examined race differences in the relationship between BMI and attractiveness, research suggests that body shape, specifically waist-to-hip ratio, is a more robust indicator of female attractiveness that is less susceptible to ethnic variations in body mass [20]. Thus, future research should examine the role of body-fat distribution in determining attractiveness perception among obese women. Also, because the data are cross-sectional, no conclusions can be made regarding the directionality of the relationship between perceived attractiveness and weight. Longitudinal research should investigate whether perceived attractiveness influences weight gain over time among obese women or vice versa, and examine whether ethnicity affects the trajectory of these variables over time. Also, it is important that the body image concerns of minority women are not overlooked on the basis of comparative research, as many Black women continue to report culture-specific body dissatisfaction. For example, Black women tend to desire a more curvaceous body type [15] and often endorse dissatisfaction with skin tone [21]. Continued research is needed on the nature of specific body image concerns among Black women.

## 5. Conclusions

In summary, the present paper provides further support for the buffering hypothesis in explaining differences in self-rated attractiveness among obese Black and White women, as Black women's evaluations of their own attractiveness were unrelated to body weight, while White women reported reduced attractiveness as their weight increased. The conclusion that Black women evaluate their attractiveness independently of perceived weight is relevant to interventions for weight loss that target this population. Further research is needed to determine the influence of body shape on perceived attractiveness among obese women and to examine whether perceived attractiveness contributes to racial differences in weight gain over time.

## Figures and Tables

**Table 1:** *t*-test results for ethnic differences in BMI, perceived weight, and attractiveness.

	Ethnicity		*t*	*df*
	Black (*n* = 531)	White (*n* = 1163)		
BMI	35.04 (8.05)	32.92 (6.59)	−5.72**	1692
Perceived weight	4.06 (.71)	4.19 (.69)	3.46**	1691
Attractiveness	3.33 (.72)	2.57 (.68)	−21.16**	1688

**p < .01. Standard deviations are in parentheses.

**Table 2:** Perceived attractiveness as a function of race.

Step	Predictor	Beta at step	*t*	*R* ^2^Δ
	(Constant)		10.75***	—
1	Age	−.034	−1.57	—
	Race	.458	21.17***	—
2	BMI	−.091	−4.19***	0.008***
3	Race × BMI	.104	3.18**	0.005**
4	Education level	.031	1.42	0.001

**p < .01 .

***p < .001.
